# Prenatal Music Exposure Induces Long-Term Neural Effects

**DOI:** 10.1371/journal.pone.0078946

**Published:** 2013-10-30

**Authors:** Eino Partanen, Teija Kujala, Mari Tervaniemi, Minna Huotilainen

**Affiliations:** 1 Cognitive Brain Research Unit, Cognitive Science, Institute of Behavioral Sciences, University of Helsinki, Helsinki, Finland; 2 Finnish Center of Excellence in Interdisciplinary Music Research, Deparment of Music, University of Jyväskylä, Jyväskylä, Finland; 3 Cicero Learning, University of Helsinki, Helsinki, Finland; 4 Finnish Institute of Occupational Health, Helsinki, Finland; University of Salamanca- Institute for Neuroscience of Castille and Leon and Medical School, Spain

## Abstract

We investigated the neural correlates induced by prenatal exposure to melodies using brains' event-related potentials (ERPs). During the last trimester of pregnancy, the mothers in the *learning group* played the ‘Twinkle twinkle little star’ -melody 5 times per week. After birth and again at the age of 4 months, we played the infants a modified melody in which some of the notes were changed while ERPs to unchanged and changed notes were recorded. The ERPs were also recorded from a *control group*, who received no prenatal stimulation. Both at birth and at the age of 4 months, infants in the *learning group* had stronger ERPs to the unchanged notes than the *control group*. Furthermore, the ERP amplitudes to the changed and unchanged notes at birth were correlated with the amount of prenatal exposure. Our results show that extensive prenatal exposure to a melody induces neural representations that last for several months.

## Introduction

Rather than being born as ‘blank slate’, a newborn has surprisingly extensive experiences on the surrounding world. In particular, newborns seem to react to sounds during the fetal period (see [1] for a review) and respond distinctly to them after birth. For example, newborns seem to recognize familiar environmental sounds [2] and melodies [3] from the prenatal environment, discriminate between the native language of the mother and other languages [4], and recognize mother's voice [5] from voices of other females. It was suggested that prenatal learning facilitates, for example, language learning in infancy [6] and provides a basis for attachment [5].

Fetal auditory learning becomes possible soon after the onset of hearing, in humans by the 27 weeks gestational age (GA) [7], when external auditory input starts to reorganize the auditory cortex [8]. Initially it was suggested that fetal auditory learning was limited to the discrimination of low-pitch sounds features, such as the rhythm of music and prosodic features of speech [9], as external high-pitched sounds are attenuated in the utero [10]. However, fetuses might perceive and recognize even the high-pitched sounds as adult listeners can recognize speech sounds when attenuated similarly as the external sounds in utero [11].

It is challenging to determine what sound features fetuses have learned prior to birth (for a review, see [12]). While behavioral measures (e.g., head-turning, non-nutritional sucking) are one possible approach, brain's event-related potentials (ERPs) can provide more specific information on the neural correlates of the types and features of sounds the fetuses can learn [13].

In adults, ERPs have been used to study both the effects of passive exposure to sounds and active auditory learning. For example, mere 15 minutes of passive exposure to sounds enhanced P2 ERP response in adults [14]. Furthermore, active auditory discrimination training enhanced P2 ERP responses and this enhancement increased after each training session, lasting months after the last auditory experience [15]. Also 1-year old infants participating in active musical training had more positive ERP responses to musical sounds than infants participating in passive musical training [16]. While newborns and fetuses cannot actively participate in learning, newborns were shown to learn during sleep [17].

The prenatal auditory learning may also be seen as an enhancement of Mismatch Negativity (MMN), a component of ERPs widely been used in studies of learning and development [18]. MMN represents the brain's automatic change-detection [18], reflects the formation of long-term memory representations (for a review, see [18]), and is elicited even in the absence of attention [19]. While in adults MMN is seen as a negative deflection within 200–300 ms from change onset in the deviant-minus-standard difference waveform, in infants MMNs of both positive and negative polarity have been reported [20]–[25]. However, the deviant-minus-standard difference waveform may also include other components, such as the positive P3a responses associated with involuntary attention shifting in adults (e.g. [26]), confounding the genuine MMN. In infancy, however, increased attention towards the sounds has been shown to elicit an additional negative component in addition to the positive MMN response in the infant deviant-minus-standard waveform while the positive response remains unchanged [27], [28]. Previous studies utilizing MMN to investigate infant auditory discrimination have shown that infants can neurally discriminate, for example, pitch changes in melodies [25].

Previously, neuroscientific research has focused on the immediate outcomes of fetal auditory learning after birth (for a review, see [12]). To our knowledge, previous follow-up studies of fetal learning have only shown cardiac effects for a melodic contour 6 weeks after birth [29]. Here we investigate with ERPs fetal auditory learning of a familiar children's' melody (Twinkle twinkle little star). Furthermore, we determine the persistency of the learning effects by following up the infants for 4 months after birth. We expected to see two possible effects due to prenatal exposure to music: a general enhancement in the ERPs to the stimuli used in the experiment, and specific learning effects seen as the elicitation and enhancement of MMR response to changed sounds in the melody.

## Methods

### Participants and ethics statement

12 Finnish- and bilingual Finnish and Swedish -speaking women with non-complicated singleton pregnancies participated in the *learning group*. In addition, a *control group* of 12 healthy newborn infants of Finnish- and bilingual Swedish and Finnish -speaking families were recruited after birth. Only one woman in both *learning* and *control groups* was bilingual. Four months later, the same infants, including the ones whose data were rejected from the initial experiment, participated in a follow-up EEG experiment. The mothers or both parents gave an informed oral consent for their infants' participation in the study. The parents received a small monetary compensation for participating in the study, and this signed transaction, using the parents' tax deduction card, was used to formally document the oral consent. In addition, the contact details of the families were taken in writing, should any need to contact the family again arise. The Ethical Committees of the former Department of Psychology and the Helsinki University Central Hospital approved both the study and the consent procedure. After removing participants due to hardware issues and excessive movement from both experiments, the final *learning group* consisted of 10 infants in the initial experiment and 10 infants at the follow-up experiment (11 and 8 infants, respectively, for the *control group*).

At birth, the hearing of the infants in both groups was tested with Evoked Oto-Acoustic Emissions (EOAE, ILO88 Dpi, Otodynamics Ltd., Hatfield, UK). All infants passed the test and were considered healthy by a neonatologist. The gestational age, birth weight, APGAR score and the age at EEG experiment are listed in Table 1. No statistically significant differences between any background variables were found between the groups either at birth or at the age of 4 months.

**Table 1:** Participant background information for the *learning* (*L*) and *control* (*C*) *groups*.

	Gestational age (weeks+days)	Birth weight (grams)	APGAR score	Age at EEG experiment (days)
Initial experiment	L: 41+0 (38+0−42+0) C: 40+3 (37+5−42+4)	L: 3834 (1975–4590) C: 3707 (3000–4700)	L: 9 (8–10) C: 9 (7–9)	L: 16 (9–27) C: 13 (2–26)
Follow-up experiment	L: 40+6 (38+0−42+0)C: 40+0 (37+5−41+6)	L: 3703 (1975–4590) C: 3831 (3345–4700)	L: 9 (8–10) C: 8 (7–9)	L: 144 (128–170) C: 133 (120–150)

Numbers denote means, the numbers in brackets denote minimum and maximum, respectively.

### Prenatal stimulation

Mothers in the *learning group* played a learning CD at loud volume at home (approximately 75 – 85 dB SPL) 5 times each week from GA 29+0 (Gestational age; weeks+days) onwards until birth and were told to destroy the CD after giving birth. The CD contained 3 short excerpts of several musical melodies, alternating with speech phrases (total duration of 15 minutes). Several melodies and speech phrases were included on the CD to make the listening more pleasant for the mother and also to capture the attention of the fetus by changes in the auditory stimulation, which might facilitate learning. One of the melodies was a 54-second long melody of “Twinkle twinkle little star”, played with a Roland A-33 keyboard in G-major and the other musical sounds on the CD were extremely different from both “Twinkle twinkle little star” and each other, being either melodies from the study of Tervaniemi et al. [30] or a classical piece by Sibelius. The mothers in the *learning group* played the CD between 46 and 64 times (mean 57). The “Twinkle twinkle little star” –melody was repeated 3 times on the CD, and the fetuses heard the melody between 138 to 192 times (mean 171).

### Stimuli and procedure

**Figure 1:**
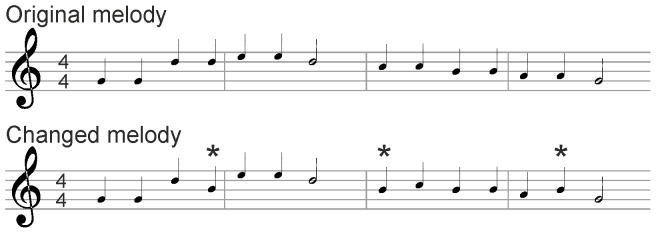
An excerpt of the stimuli used in the experiment. The image score above represents the original unchanged melody while the score below shows the changed melody. Changed notes are marked with asterisks.

In the EEG-experiments a modified version of the “Twinkle twinkle little star” -melody was played to the infants 9 times. In the modified melody, 12.5% of the notes in the original melody were replaced at random with B (H in German notation) -notes (called *changed sounds* from now on; the unchanged notes are referred to as *unchanged sounds; see* *Figure 1*). The changes are key-preserving as B belongs to G-major scale and thus are not more salient than unchanged notes on basis of the key of the melody. However, a listener who is familiar with the original melody can recognize the changed sounds easily (see, e.g., [31], for a similar paradigm). The melody was played with a 600 ms stimulus onset asynchrony (SOA) between the sounds. With the exception of the changed sounds, all characteristics of the experimental melody (e.g. tempo, key) were kept identical with the melody the infants were exposed to prenatally. Speech phrases and other musical sounds, similar to those on the learning tape, were presented between the melodies.

During the newborn recordings, sounds were presented via loudspeakers 20 cm to the left and to the right from the infant's head. In the four months follow-up recording the loudspeakers were located one meter to the left and to the right of the infant due to the infants being awake and possibly grabbing the loudspeakers if they were placed too close to the infant. The sound intensity was approximately 70 dB (SPL) at the infant's head in both recordings.

### ERP recording and data analysis

The newborn ERP recordings were conducted by trained nurses at the Hospital for Children and Adolescents, Helsinki University Central Hospital and the four-month follow-up recordings at the Cognitive Brain Research Unit, University of Helsinki. Disposable EEG electrodes were placed at F3, F4, C3, Cz, C4, P3, P4, T3, and T4 scalp loci and the two mastoids according to the international 10–20 system (T3 and T4 electrodes were omitted from the four-month follow-up recording due to lack of signal in the newborn recordings). EOG was monitored with electrodes below and to the right of the right eye. EEG was recorded with a sampling rate of 250 Hz through a band-pass filter of 0.1 to 40 Hz using the NeuroScan recording system, referenced to the average of the mastoid electrodes. During the recording the infants were laying in a crib and the four-month-olds were either placed in an infant car seat or in their parents' or nurses' lap.

The sleep stage of the infant was classified to be active (active sleep, AS) when the EEG showed low-voltage high-frequency activity, quiet (quiet sleep, QS) when it included either high-voltage low frequency activity or trace alternants (high and low-voltage slow waves alternating) and awake when the EOG channels showed frequent and large eye movements or large movement artefacts and on basis of the observations of a trained nurse conducting the experiment (see, e.g., [32], for classification criteria). The EEG data during which the infant was awake (even for an occasional period of time), or with large artifacts, were rejected during the visual analysis of the sleep stages

Offline, all the data were analyzed in sensor space. Initially the data from newborns were first divided into sleep stages, determined using the EEG, EOG, and the notes from the nurses. AS is characterized by low-voltage high-frequency activity, QS by either high-voltage low frequency activity or trace alternants (high and low-voltage slow waves alternating), while during wakefulness EOG channels show frequent and large eye movements or EEG channels large movement artifacts [32]. The newborn wakefulness was determined both on the basis of EEG and of the observations of a trained nurse conducting the experiment. Because of extensive artifacts, the data recorded during the infant's wakefulness were discarded from further analysis. The data collected during active and quiet sleep were combined. The infants in the *learning group* spent 0–100% (mean 64%) of their time in quiet sleep (27 – 100%, mean 73%, for the *control group*), measured by the number of accepted epochs in quiet sleep versus accepted epochs in active and quiet sleep combined. By four months of age, the infants sleep much less during the day than newborns do [33], and the four-month old infants spent most of the experiment awake. Unlike newborns, the four-month-olds did not move extensively during the recording when awake, and thus these data were used as well, after removing any data with movement artifacts.

During data analysis the two stimuli immediately following a changed sound were discarded from the analysis. The data were offline-filtered with a zero-phase band-pass filter (1 to 20 Hz) and divided into epochs of 700 ms starting 100 ms prior to sound onset. T3, T4, P3, and P4 electrodes were removed from further analysis due to lack of signal. All epochs including movement artifacts or those in which the amplitude on any of the channels exceeded ±100 µV were excluded from further analysis. The epochs for the unchanged and changed sounds were separately averaged. To study MMRs, difference signals were formed by subtracting the response to the unchanged sound from that to the changed sound. Group-average signals were formed for unchanged and changed sounds, and for difference signals, separately for both *learning* and *control groups*. To improve the S/N ratio, the signals from F3, F4, C3, Cz, and C4 electrodes were averaged together.

ERP and MMR peak latencies were determined from the group-average waveforms, separately for both groups and both experiments. During the first year of life, unlike in adults, the most salient component in the auditory ERP waveform is P350 response [21], [34]. To assess P350 for the newborns, the latency of the most positive peak in the group-average waveforms between 100 and 600 ms was selected for analysis. In the four-month-olds, the responses for the unchanged and the changed sounds between 100 and 600 ms showed two positive peaks, possibly corresponding to P150 and P350 [21], both of which were analyzed further. In newborns, the difference waveform showed a single positive deflection while in four month olds the difference waveform consisted of a low-amplitude negative deflection followed by a positive peak (see also [35]), all of which were separately analyzed.

After determining the peak latencies, the mean ERP and MMR amplitudes were calculated as a mean voltage in a 60-ms window centered at the peak latency in the group-average signal. To determine whether ERPs and MMRs were statistically significant, the mean amplitudes were compared to zero using two-tailed t-tests, separately for both groups. Two-tailed t-tests were used to compare responses between the *learning* and *control groups*. Levene's test was used to assess the equality of variances and corrected t-values were used in cases of unequal variances. Effect sizes (Cohen's *d*) were calculated for all between-group comparisons. Pearson correlation was used to study whether the number of times the infants had heard the melody affected response amplitudes. For correlations, coefficients of determinations (R^2^) are reported.

## Results

**Figure 2:**
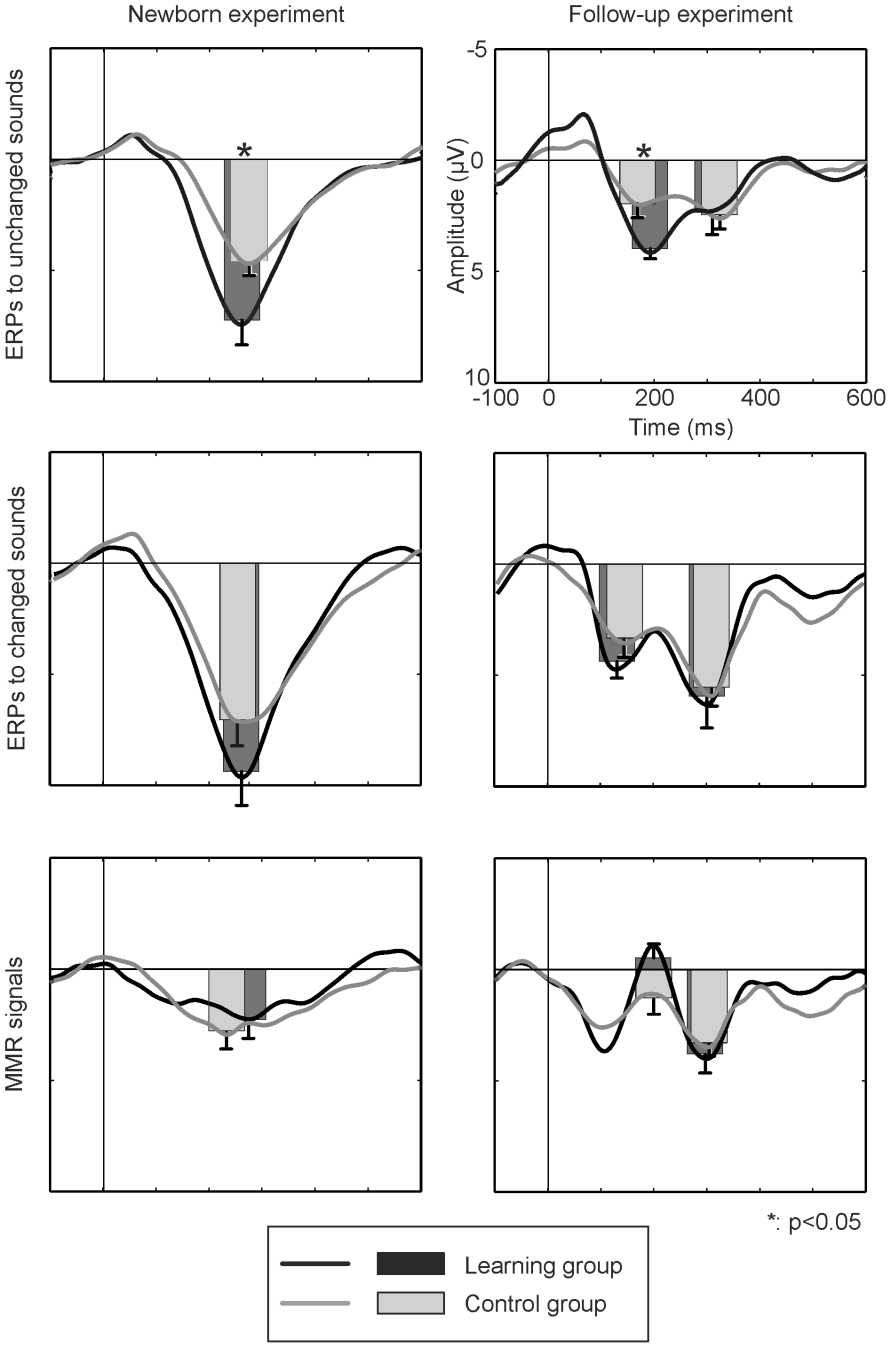
ERP and MMR amplitudes in both *learning* (dark bars) and *control groups* (light bars) at birth (left) and at the age of four months (right). Responses to unchanged sounds were stronger in the *learning* than *control group* at birth and at four months of age. Asterisks denote statistical significances, error bars denote standard errors of the mean.

In newborns and at the age of 4 months, statistically significant ERPs (see Figure 2, upper and middle panels, and Table 1) were elicited by all sounds in both groups, with the exception of the late peak to the unchanged sounds in the *learning group* at the age of 4 months, which only tended to be statistically significant. Positive MMRs to changed sounds between 200 and 300 ms after stimulus onset were statistically significant both in newborns and at the age of 4 months in both groups (see Figure 2, bottom panels, and Table 2). The negative MMR peak in 4 month olds was not statistically significant.

**Table 2:** Statistical significances of the ERP and MMR responses for the *learning* (*L*) and *control* (*C*) *groups* both at birth and at the age of 4 months.

Experiment	ERPs to unchanged sounds		ERPs to changed sounds		MMR
Newborns	*L*: t(9) = 6.582*** *C*: t(10) = 6.827***		*L*: t(9) = 6.100*** *C*: t(10) = 6.144***		*L*: (t(9) = 2.610* *C*: (t(10) = 3.279**
4-month olds	Early peak *L*: t(9) = 7.988*** *C*: t(7) = 6.173***	Late peak *L*: t(9) = 2.191 *C* t(7) = 3.850**	Early peak *L*: t(9) = 6.475*** *C*: t(7) = 3.987**	Late peak *L*: t(9) = 4.280** *C*: t(7) = 8.013***	*L*: t(9) = 4.277** *C*: t(7) = 5.095***

Asterisks denote statistical significances.

* : p<0.05, **: p<0.01, ***:p<0.001.

Between-groups comparisons showed that the responses to the unchanged sounds were larger in the *learning* than *control group* both at birth (t(19) = 2.11, p<0.049, *d* = 0.97) and at the age of four months (t(16) = 3.33, p<0.004, *d* = 1.68). Furthermore, a correlation was found showing that the more often the newborns had heard the learning CD, the larger the amplitudes to the unchanged (r = 0.74, p<0.015, R^2^ = 0.54) and changed sounds (r = 0.68, p<0.032, R^2^ = 0.46) were. This effect was no longer seen in the follow-up experiment (p>0.22 for all tests). For MMR amplitudes, no group differences were found.

## Discussion

We investigated the formation and retention of neural representations induced by exposure to melodies during the fetal period. At birth the *learning group* had larger ERPs to the melodies they heard as fetuses than the *control group* for whom the melodies were unfamiliar. This difference was still significant at the age of 4 months. Furthermore, ERP amplitudes for unchanged and changed sounds at birth were correlated with the number of times the infants in the *learning group* were exposed to the melody as fetuses. These results show that fetal exposure to melodic sound patters can form neural representations which last for several months. Our results also suggest that the effects of prenatal exposure are much more long-lasting than reported in the very few studies conducted previously, which have shown effects from prenatal exposure lasting for at least six weeks for a melodic contour [29] with no additional stimulation after birth.

The larger ERPs in the *learning* than *control group* cannot be exclusively explained by possible inborn differences in auditory processing since ERPs to the changed and unchanged sounds were correlated with the amount of prenatal exposure. The correlation effects support the findings of previous studies on learning which have shown infants and newborns to be extremely fast learners, capable of learning, for example, statistical regularities of sounds in 2 minutes of stimulation [36]. Furthermore, mere 15 minutes of unattended exposure to unchanged sounds enhanced the ERP responses for those sounds in adults [14], [37]. However, as the amount of exposure was correlated with both responses to changed and unchanged sounds, it may also reflect a nonspecific effect of music on auditory processing instead of learning of specific sounds of the melody pattern.

We also found that both the *learning* and *control groups* had statistically significant MMRs to changed sounds in the melody. Unlike the ERPs for unchanged sounds, the MMR amplitudes did not differ between the groups. However, in adults the effects of musical training on MMN are usually seen after active listening to the melodies, not merely after exposure to melodies [38], and only a modest enhancement of P2 response amplitude has been shown after passive musical exposure to those sounds [39], [40]. Thus, while the exposure to melodies may modestly enhance ERP responses for those sounds, active sensorimotor training seems to be much more efficient in inducing these changes [39] and such training might be required for MMR to be enhanced. Alternatively, the statistically significant MMRs elicited by both groups may reflect merely physical difference between the changed (all B-notes) and unchanged notes (mostly other than B-notes).

Taken together, our results show that prenatal exposure to music can have long-term plastic effects on the developing brain and enhance neural responsiveness to the sounds used in the prenatal training, an effect previously only demonstrated in animal models [41]. Furthermore, we found that these plastic changes are long lasting, as the effect of prenatal exposure persists for at least four months without any additional stimulation. These findings have several practical implications. First, since the prenatal auditory environment modulates the neural responsiveness of fetuses, it seems plausible that the adverse prenatal sound environment may also have long-lasting detrimental effects [8]. Such environments may be, for example, noisy workplaces and, in case of preterm infants, neonatal intensive care units. Furthermore, as prenatal exposure still affected the ERP responses months after birth, additional fetal exposure to structured sound environments might be beneficial for supporting the auditory processing of, for example, infants at risk for dyslexia in whom basic auditory processing was shown to be impaired (e.g., [42]). Such effects have previously been demonstrated in rat pups, showing benefits of structured sound environments during pregnancy for cortical organization and synaptogenesis [41], and enhancing their spatial learning ability for up to 21 days after birth [43]. However, further studies are needed to shed light on the specific mechanisms of enhanced neural responsiveness induced by the prenatal stimulation, and to determine whether such stimulation could be used to alleviate the deficits in auditory processing.
